# The Ex-Premier and the Poet

**Published:** 1891-06-13

**Authors:** 


### Passing Topics.

### THE EX-PREMIER AND THE POET.

The author of the small volume of poems, "By the Sea," has well merited the rebuke he has drawn upon himself from no less a personage than Mr. Gladstone, and we are almost induced to regret that the admirable lesson read this aspiring poet should have been tempered by a single line of commendation. For it seems to us there is nothing in all the wide range of wrong more wrongful, and nothing in all the wide range of snobbery more snobbish and impertinent, than to cast scorn upon the worker for taking a workman's wage honestly earned. And when the wrong and the snobbery are wedded to verse, more or less immortal, the critic would be wanting, indeed, if he did not seek to divorce the error. Why even poets, we mean those who can boast the real afflatus, are not one whit more unready than the rude multitude to grasp their rightful gains; and, doubtless, the writer of "By the Sea" would not object if the tide chanced to run in upon a golden strand, and is fully prepared to accept such returns as the sale of his now well-advertised work may afford.

Mr. Gladstone's rebuke appears almost too mild when he begs the poet will "forgive me if I expostulate a little on behalf of the hired priest, whom you find it necessary to scorn." The hired priest of all people! Our poet has not even the merit of courage, or the hired premier might have suggested itself to him. The declaration which follows, that "if the priest is to live, he must beg, earn, or steal," is irresistible, and no doubt the poet's sense of discrimination is equal to answering the inquiry, "Which is best?" But Mr. Gladstone goes further: he illustrates his argument aptly enough, but, as many people will think, quite unnecessarily. "Within the last six months," he writes, "three at least of the most distinguished men of Oxford, Dean Church, Canon Liddon, and Mr. Aubrey Moore, have, I believe, shortened by devoted labour their most valuable lives. But all were hired priests." The hearty good wishes with which this remarkable epistle ends ought certainly to cover, as it was no doubt intended they should, a larger share of that correct taste and sweet reasonableness of mind which writers of the style under notice are so frequently deficient in.

It is unfortunate that the author of "By the Sea" is not alone in his scorn for "hired priests," or in fact, for anyone who is wholly or in part paid for doing good and useful work. A man may devote all his strength and his ability to amassing wealth, and if he succeed, he is a respectable and important member of society. Nobody, least of all poets in search of a patron, will think of levelling reproach at him. His industry is commended, and his example is held up to imitation. But in the case of the man who works for the good of his fellows, let him receive ever so inadequate a wage, and at once to a certain, and it must be confessed, a fairly numerous class of minds, he becomes little short of a hypocrite, and is held to be actuated not in the minutest degree by love of his kind or of his work, but altogether by the thought of his emoluments.

It is just as well the world should be reminded occasionally that even obscure versifiers take responsibility when they launch their volumes. It is not necessary to pour broadsides into cockleshells, nor is to be expected that every poetaster's venture will receive the attention of an ex-premier, but mischievous thoughts, though they cannot live upon the great seas, may float awhile in backwaters and eddies, and it is best they should be sent quickly to the bottom.

Agricola.

